# Professor Wolfgang Grisold, President of the World Federation of Neurology: Adapted Interview from the 12^th^ World Congress for NeuroRehabilitation (WCNR) – Vienna, 2022

**DOI:** 10.25122/jml-2023-1004

**Published:** 2023-01

**Authors:** Alexandra Gherman, Stefana-Andrada Dobran

**Affiliations:** 1 RoNeuro Institute for Neurological Research and Diagnostic, Cluj-Napoca, Romania; 2 Sociology Department, Babes-Bolyai University, Cluj-Napoca, Romania

**Interviewer: Alexandra Gherman**

**Interviewee: Professor Wolfgang Grisold**

Professor Grisold began his four-year term as President of the World Federation of Neurology (WFN) on 1^st^ January 2022, making him the first Austrian to accede to the WFN presidency. Professor Wolfgang Grisold was also the very first president of the Austrian Society of Neurology, elected in 2000. His focus is on general neurology, neuro-oncology, neuromuscular disease, palliative care, and education. In these fields, he has contributed to over 200 peer-reviewed articles; he also published many books ^1^ and participated in several EU projects. Professor Grisold has always been interested in education-related work. He has been part of several education committees and also chaired the development of the "European board examination" and the WFN teaching course committee. He is also involved in European CME (Continual Medical Education) accreditation.

**A.G.:** Professor Grisold, we are here, in Vienna, for the 12^th^ World Congress for Neurorehabilitation organised by the World Federation for NeuroRehabilitation. What is your first-hand opinion so far, and how many previous editions have you taken part in?

**W.G.:** Thank you very much for asking! I think it's a great idea that this large congress, with two thousand or three thousand people, takes place in Vienna. I have watched the rise of the World Federation for NeuroRehabilitation over the years, and it started as a small congress – I think the first congress was in Vienna, and several Austrians were involved, in particular, the late Prof. Gerstenbrand.

**A.G.:** Yes, I believe so.

**W.G.:** Neurorehabilitation it's getting to be a big and important movement. I think one of the advantages of this organisation is that it is multidisciplinary and also multi-professional, and it also reflects real life, where not only neurologists see patients with neurological diseases, but there are many more care professions. I think it's one of the big advantages of the congress. I've just been attending an international session that was jointly organized with the World Federation for NeuroRehabilitation and the World Federation of Neurology (*WFNR and WFN Joint Symposium – Approaches to Brain Health*), and there were many ideas spread. I think, looking at the regions of the world, that there is a lot to do in order to get joint concepts.

**A.G.:** Thank you very much for your answer! What is the impact of such events on the new generation of specialists in neurorehabilitation?

**W.G.:** The structure of the organisations is changing, and we have all seen the change from the hierarchic structures down to the more bottom-up structures. I think in many organisations young neurologists have a significant role already, but I believe it will still be a matter of discussing, of implementing, and using the forces that are created. By that, I mean forces of young neurologists who have good and fresh ideas and some of them may not be conventional, giving stimuli to development. I think – and this is a compliment to the WFNR, that such a platform which allows the presence of young people, is a creative platform enabling new avenues of development. I was just attending a meeting before with another medical society, who was complaining that in the age group of 40 to 50 years, at present, they have no leaders because, for some reason, they forgot to produce leaders. The American Academy of Neurology, for example, has had leadership programs for years. It is important to teach leadership and make young neurologists aware that seeing patients, interacting with staff, with other disciplines, and with carers needs a leadership. And we have to make young people aware of that responsibility.

**A.G.:** Yes, you are right. Thank you very much for your answer! As the President of the World Federation of Neurology, what do you consider the primary organisational gaps in neurorehabilitation at the international level? I think this connects with your previous answer.

**W.G.:** It connects with my previous answer, and having been to this session (*WFNR and WFN Joint Symposium – Approaches to Brain Health*) where I've been just now, I'm more concerned than before because, obviously, there are only few countries where this concept of neurorehabilitation seems to be implemented. German, Swiss, and Austrian societies for years have been advancing on neuro rehabilitation in their countries. And if you look at this concept of rehabilitation centres, I mean, in particular, in Germany, where this works so fast and connected and is usually or controlled by neurologists who are dedicated to neurorehabilitation – this is an excellent template. In other countries of Europe, I think there are similar developments, but I think this organisational structure, from acute disease to implementing rehabilitation into the disease trajectory, is best developed in stroke. For some countries, in particular in Africa, there are many health issues to solve before neurorehabilitation can be implemented, and of course, this is resource dependent. As an example, I have only recently spoken to a young colleague from an African country who said: "Well, the [patients] go to the stroke unit", "And then?", "Well, they go back to the families", meaning implicitly that neurorehabilitation is left to the abilities of the family, and professional help is often missing.

**A.G.:** Yes.

**W.G.:** I have also been very engaged in neuro-oncology. In the past, if you tried to send a patient with a brain tumour to a rehab centre, they would just refuse, or neuromuscular disease, ALS (Amyotrophic Lateral Sclerosis) patients for the same reasons. But I think there is a big change. And the change that happened is that rehabilitation does not only mean cure. Rehabilitation can help to gain functions of daily living and quality of life, meaning a lot to the patients.

**A.G.:** How can primary and tertiary prevention be improved, concerning neurological diseases, especially for low- and middle-income countries?

**W.G.:** Prevention is something that we learn about, we know about, and we do it in some ways, but, of course, it can be widely increased. I think one of the best examples is stroke mortality in Europe. In particular, in Eastern Europe, the management of hypertension and the availability of treatment have shown that it makes a big difference. Yet, many people in the world are not aware of that. This is valid for the most frequent disease: stroke. There are many other examples, as epilepsy is, where you would be able to prevent head trauma. There are many examples of how prevention can be done. From my experience, having also seen many patients in a public practice, not in a high-level tertiary centre, people are not aware of that. As we face many neurological diseases, several concepts, and strategies have to be used for effective prevention. Concepts for awareness and prevention have been demonstrated previously by cardiology, and neurology needs to follow the same paths. I also want to remind here that there are some factors we cannot influence. One factor is genetics, another one is age. And I want to emphasize how important it is for neurology to fight ageism. People should receive treatment and the best care, also in old age.

**A.G.:** Yes, it is, and it is connected to the quality of life. Because being old does not mean that you necessarily have a low quality of life, for example.

**W.G.:** This is, by all means, implicit. The quality of life should be preserved in all circumstances. Of course, we know that several aspects, as social factors and personal factors, change over time. Also, multimorbidity may be an important factor to consider.

**A.G.:** Yes, thank you very much! How can multidisciplinary, international collaboration be strengthened to provide the best evidence and care for patients?

**W.G.:** One of the problems is that specialists often do not think outside of their specialty. To include other fields and considerations is important in care. The idea is that specialties, although different, should be able to connect. I think this WFNR and this Rehabilitation Congress are a good template that various fields can work together. The management of diseases is varying in many countries, and cultural aspects also have to be considered. In several fields of neurology, the interdisciplinary and multi-professional aspects are well developed. Neurology, other medical fields, nursing, physiotherapy, social work, and often the carers need to be involved in therapeutic concepts.

**A.G.:** So, basically, one needs to think a little bit more outside the box as a specialist and as a caregiver, right? Would you agree?

**W.G.:** Yes, thinking out of the box is important, and it is our task to think out of the box. We must be aware that the concept of medicine, as it is now, has many incentives that often do not encourage us to think out of the box. Roadblocks are hospital costs, insurance wishes, age limits, and others. As neurologists, we need to be aware that we are the advocates of patients and caregivers, and we are not the advocates of hospital managers or health care plans. The return of medical management responsible for hospital management and health management is important. We need to be as economic as possible, but the purpose it's not to save on health; it is that of giving the best health care affordable to the patient.

**A.G.:** Yes, thank you very much, Professor Grisold, and best of luck in all your endeavours!

**W.G.:** Thank you, I need it! Thank you!

## Footnotes

1 Electromyography (eds. Mamoli,B., Caruso,G., Grisold,W.):Facultas Universitätsverlag, Wien, 1987, 95-102. Brainlinc, Deutsche Ausgabe 3, Hartung, Barahona, Garcia, Grisold W, Kesselring, Montalbano, Munschauer, Chris, Poser Urban & Fischer, ISBN 978-3-437-41478-7. Neuromuskuläre Läsionen bei Malignomen. Springer © 1989 Autor: W. Grisold. Praktische Neuroonkologie, Springer © 2000 Autoren: Grisold Wolfgang, Krauseneck, Peter, Müller, Bettina. Atlas of Neuromuscular Diseases (Ed 1), 2006 A Practical GuidelineFeldman E, Grisold W, Russel J, Zifko U. Neuro-Oncology Part I 2012. Handbook of Clinical Neurology (Series Editors: Aminoff, Boller and Swaab), von Grisold, Wolfgang, Soffietti, Riccardo Hrsg. Neuro-Oncology, Part II 2012. Handbook of Clinical Neurology (Series editors: Aminoff, Boller, Swaab), von Grisold, Wolfgang, Soffietti, Riccardo Hrsg. Atlas of Neuromuscular Diseases (Ed 2), 2016 A Practical Guideline - Previously published in hardcover (ed 2), Feldman E, Grisold W, Russel J, Löscher W. Atlas of Neuromuscular Diseases (Ed 2), 2016 (Russisch) A Practical Guideline - Previously published in hardcover, Feldman E, Grisold W, Russel J, Löscher W, ISBN 978-3-7 091-1604-3 (anr.r..), ISBN 978-5-98811-382-9 (pyc.) Advocacy in Neurology 2019, Grisold W, Struhal W, Grisold Th, OUP Oxford, Grisold W, Berlit P. Aktuelle Therapie in der Neurologie. (Lose Blatt Werk), Laufend bis 2021, Ecomed, Medizin, Feldman R, Russel JW, Löscher W, Grisold W, Meng S. Atlas of Neuromuscular Diseases (Ed 3), A Practical Guideline - Springer, ISBN 978 3 030 63448 3, 2021, Grisold W, Soffietti R, Oberndorfer S, Cavaletti G. Effects of cancer treatment on the nervous system Vol 1 (Cambridge Scholars) Vol 1, 2020, ISBN (10) 1 5275 5888 6, Grisold W, Soffietti R, Oberndorfer S, Cavaletti G. Effects of cancer treatment on the nervous system Vol 2 (Cambridge Scholars) Vol 2, 2021, ISBN (10) 1 5275 6087 2. Neurology in Migrants, Springer Nature, Sustainable development goals, ISSN 2523-3084, 2021. Polyneuropathie and Fallbeispielen. Kohlhammer 2022, Lehmann H., Grisold W.

